# A new member of the psToc159 family contributes to distinct protein targeting pathways in pea chloroplasts

**DOI:** 10.3389/fpls.2014.00239

**Published:** 2014-05-28

**Authors:** WaiLing Chang, Jürgen Soll, Bettina Bölter

**Affiliations:** ^1^Department Biology I, Plant Sciences, LMU MünchenMartinsried, Germany; ^2^Munich Center for Integrated Protein Science CiPSMünchen, Germany; ^3^Lysando GmbHRegensburg, Germany

**Keywords:** chloroplast, import, receptor protein, targeting, pea

## Abstract

Protein import into chloroplasts relies on specific targeting of preproteins from the cytosol to the organelles and coordinated translocation processes across the double envelope membrane. Here, two complex machineries constitute the so called general import pathway, which consists of the TOC and TIC complexes (translocon at the outer envelope of chloroplasts and translocon at the inner envelope of chloroplasts, respectively). The majority of canonical preproteins feature an N-terminal cleavable transit peptide, which is necessary for targeting and recognition at the chloroplast surface by receptors of TOC, where Toc159 acts as the primary contact site. We identified a non-canonical preprotein without the classical transit peptide, the superoxide dismutase (FSD1), which was then used in chemical crosslinking approaches to find new interaction partners at the outer envelope from pea chloroplasts. In this way we could link FSD1 to members of the Toc159 family in pea, namely psToc132 and psToc120. Using deletion mutants as well as a peptide scanning approach we defined regions of the preprotein, which are involved in receptor binding. These are distributed across the entire sequence; however the extreme N-terminus as well as a C-proximal domain turned out to be essential for targeting and import. En route into the plastid FSD1 engages components of the general import pathway, implying that in spite of the non-canonical targeting information and recognition by a specific receptor this preprotein follows a similar way across the envelope as the majority of plastid preproteins.

## Introduction

Plastids represent a large set of organelles with distinct physiological functions and morphologies found within all plant cells (Lopez-Juez and Pyke, 2005). The best studied plastid type are chloroplasts—photosynthetic organelles in plants and green algae that are responsible for converting energy from sunlight into carbohydrates and ATP. They are surrounded by a double membrane and possess their own genome, although the vast majority of genes encoding for chloroplast proteins has been transferred to the nucleus in the course of evolution. Analysis of the chloroplast genome revealed that the origin of chloroplasts can be traced back to a cyanobacterial ancestor that was engulfed by an ancient eukaryotic cell and eventually integrated as an organelle during evolution. To ensure a return passage of the proteins encoded by the relocated genes back to their compartment of origin where they carry out their functions, the development of a post-translational protein trafficking system became necessary. This post-translational protein import is mainly achieved by two multimeric protein complexes (also known as the general import complexes) located at the outer (TOC—Translocon at the Outer envelope of Chloroplasts) and inner (TIC—Translocon at the Inner envelope of the Chloroplasts) envelopes of chloroplasts, respectively (Seedorf et al., 1995; Jarvis, 2008). For many years, all proteins destined to the internal chloroplast compartments were believed to possess an N-terminal chloroplast targeting sequence (also known as the transit peptide), and to engage the TOC/TIC machinery. Toc75, Toc159, and Toc34 were among the first components of the chloroplast import machinery to be identified in pea chloroplasts (Schnell et al., 1994). Toc75 is a β—barrel protein constituting the protein translocation channel in the outer envelope membrane (Schnell et al., 1994; Hinnah et al., 1997; Keegstra and Cline, 1999). The receptor components Toc159 and Toc34—both associated with Toc75—are integral proteins at the outer membrane regulated by GTP binding and hydrolysis (Kessler et al., 1994; Hirsch and Soll, 1995; Seedorf et al., 1995). They belong to a superfamily of GTPases, characterized by a soluble GTPase domain exposed to the cytosol (G-domain) and a membrane anchoring domain (M-domain). The G-domains of the TOC GTPases exhibit classical motifs of nucleotide binding and hydrolysis characteristic to that of many GTPases. It is generally accepted that the TOC GTPase receptors control transit peptide recognition and the initial stages of membrane translocation by GTP-binding and -hydrolysis which lead to molecular rearrangements, but the molecular details that hinges GTPase activity with receptor function remain need to be further investigated. In addition to these two domains, Toc159 harbors an N-terminal region, which is highly acidic (A-domain) (Kessler et al., 1994). These GTPases are unique to plastids and are responsible for recognition of nuclear–encoded precursor proteins at the outer envelope. Together, Toc159, Toc34, and Toc75 form a stable core TOC complex sufficient for precursor protein translocation into artificial liposomes *in vitro* (Schleiff et al., 2003). Although most of the components of the import machinery were originally identified in pea chloroplasts, homologs are reported in moss (*Physcomitrella patens*) as well as in all higher plant species analyzed (Kalanon and McFadden, 2008). In some of them several components (particularly constituents of the TOC core complex) build multi-gene families. For instance, the Arabidopsis *(Arabidopsis thaliana)* genome encodes two paralogs of Toc34 (atToc33 and atToc34) (Jarvis et al., 1998; Gutensohn et al., 2000) and four paralogs of Toc159 (atToc159, atToc132, atToc120, and atToc90) (Bauer et al., 2000; Hiltbrunner et al., 2004; Ivanova et al., 2004; Kubis et al., 2004) and Toc75 (atToc75-III, atToc75-IV, atToc75-I, and atToc75V/atOEP80) (Baldwin et al., 2005). The presence of different isoforms of the core TOC complex members might allow remodeling of the import machinery in accordance to the biochemical requirements of the plastid dependent on the developmental stage and/or environmental conditions. psToc159 is most similar to atToc159 (with a sequence identity of 48%). Therefore, these two proteins are believed to be functional orthologs (Bauer et al., 2000). Characterization of the T-DNA insertion mutant of atToc159, *ppi2* (*plastid protein import 2*), showed that the differentiation of proplastids into chloroplasts was arrested, resulting in an albino phenotype (Bauer et al., 2000), i.e., the plants cannot develop photoautotrophically. The accumulation of photosynthesis-related proteins was found to be drastically decreased in the *ppi2* mutant, which was not the case for non-photosynthetic plastid proteins. This led to the proposal that import of non-photosynthetic proteins is mediated by other members of the atToc159 receptor family, namely atToc132 and atToc120 (Bauer et al., 2000; Kubis et al., 2004). These different receptors indeed assemble into structurally distinct translocation complexes that exhibit unique preprotein binding properties different to atToc159-containing complex (Bauer et al., 2000; Ivanova et al., 2004; Kubis et al., 2004). The three paralogs share high sequence similarity in their M- and G-domains, but are more dissimilar in the A-domain. Upon swapping of the A-domains the selectivity of the different receptors is altered (Inoue et al., 2010). While the presence of the A-domain clearly plays a role in determining substrate specificity, it is however not essential for plant survival (Lee et al., 2003).

Although only a few proteins have been described to use alternative translocation pathways so far, these examples nevertheless indicate that the TOC/TIC machinery is not exclusively responsible for transport of proteins into plastids. Both atToc132 and atToc120 were found to form a single TOC complex together, distinct from atToc159 (Ivanova et al., 2004). atToc33 was found to co-immunoprecipitates predominantly with atToc159, whereas atToc34 forms a complex together with atToc132/tToc120 (Ivanova et al., 2004). This observation led to the notion that the core TOC complex in Arabidopsis comprises either atToc159/33/75 or atToc132/120/34/75, where specificities are reflected by their individual receptor diversities (Ivanova et al., 2004). In both complexes, only one functional Toc75 homolog (atToc75-III) was detected (Ivanova et al., 2004).

Though distinct TOC complexes have so far been only reported in Arabidopsis, the presence of multiple genes encoding distinct TOC components can also be observed in other species. Bioinformatic analysis demonstrates the diversity in TOC receptors in *Spinacia oleracia* (spinach) (Voigt et al., 2005), *Oryza sativa* (rice) (Kubis et al., 2004), *Physcomitrella patens* (Hofmann and Theg, 2004) and *Picea abies* (spruce) (Fulgosi and Soll, 2002). The existence of the structurally and functionally distinct TOC complexes might therefore function as adaptation strategy during plastid differentiation in plants. In this context, multiple import pathways could allow efficient targeting and import of both ‘housekeeping’ and light regulated or other proteins which are required for specialized metabolic functions in the dynamic life of plastids (Inaba et al., 2005).

Recent studies of the Arabidopsis chloroplast proteome revealed the existence of several “non-canonical” chloroplast proteins, suggesting that they enter chloroplasts in an TOC/TIC—independent manner via internal, non-cleavable targeting sequences (Miras et al., 2002; Nada and Soll, 2004; Zybailov et al., 2008). We re-assessed the published data and chose several proteins strongly predicted to lack a cleavable transit peptide. By analyzing the *in vitro* import behavior of this subset of putative non-canonical proteins we found that most of them do feature cleavable targeting sequences in contrast to the *in silico* predictions. We could identify only a single protein with distinct import characteristics, which was then used as a bait to identify interacting components at the outer envelope membrane by chemical cross-linking. Thereby, we identified a new member of the Toc159 family in pea.

## Materials and methods

### Plant material

Pea plants (*Pisum sativum* var. Arvica) were grown under a 16 h light/8 h dark regime at 21°C. For chloroplast isolation, plants were harvested after 9–11 days from the dark (in the morning before the lights came on). Arabidopsis was grown under long day conditions with 100 μE at 20°C.

### Construction of plasmids

All genes for the import substrates were amplified from Arabidopsis cDNA with primers containing suitable restrictions sites. The PCR products were purified via a NucleoSpin Extract II kit (Macherey-Nagel, Düren, Germany) and submitted to restriction digest. These were ligated into restricted pSP65 vector. For heterologous overexpression, the cDNAs encoding FSD1 and psToc120A were cloned into pET21b. For generation of deletion constructs the FSD1/pSP65 plasmid was used as template with appropriate oligo nucleotides (see Supplemental Table 1).

### RNA extraction, RT- and race-PCR

Leaves from 5 to 7 days old pea plants were ground in liquid nitrogen and total RNA was isolated applying the RNA Easy Isolation kit (Qiagen, Hilden, Germany) according to the manufacturer's recommendation. cDNA was prepared from 1 μg DNase-treated RNA using the BD SMART™ RACE cDNA Amplification Kit (Clontech, Germany). RT-PCR and 5'-RACE PCR were performed according to the manufacturer's instructions (BD SMART™ RACE cDNA Amplification Kit, Clontech, Germany).

### Transcription and translation

Transcription was performed in a final volume of 50 μl in the presence of SP6 RNA polymerase. The mixture included polymerase buffer, 100 U RNase inhibitor, 10 mM DTT, 25 μM BSA, 2.5 mM m^7^ GpppG, 2.5 mM each ATP, CTP, UTP, and 1 μg linearized plasmid. After 15 min of incubation at 37°C, 1.2 mM GTP was added and RNA synthesis was continued for 2 h at 37°C. The synthesized mRNA was used for translation in a final volume of 100 μl. Flexi Rabbit Reticulocyte Lysate (Promega, Madison, USA) was supplemented with RNase inhibitor, amino acids without methionine, [^35^S]methionine/cysteine (5.4 MBq; PerkinElmer, Rodgau, Germany) and potassium acetate. The translation mixture was incubated for 1 h at 30°C and centrifuged at 50,000 × g for 20 min at 4°C. The supernatant was used for all import experiments. In some cases ATP was depleted from the translation product by using Micro Bio-Spin Chromatography Columns with Bio-Gel P-6 in Tris Buffer (Biorad, Munich, Germany) according to manufacturer's recommendations.

### Isolation of intact chloroplasts and protein import experiments

Chloroplasts were isolated from 9 to 11 day old pea plants according to Waegemann and Soll (1996). Concentration of chlorophyll was determined as described (Arnon, 1949). Intact chloroplasts were incubated 30 min on ice in the dark to deplete ATP. A standard import reaction contained chloroplasts equivalent to 10 μg chlorophyll in 100 μl import buffer (330 mM sorbitol, 50 mM HEPES/KOH pH 7.6, 3 mM MgSO_4_, 10 mM methionine, 10 mM cysteine, 20 mM K-gluconate, 10 mM NaHCO_3_, 2% BSA (w/v) and 3 mM ATP (unless indicated otherwise) and up to 10% of [^35^S]-labeled precursor protein. The import reaction was conducted for 15 min at 25°C unless indicated otherwise. Chloroplasts were re-isolated by centrifugation through a 40% Percoll cushion and washed twice in wash medium (330 mM sorbitol, 50 mM HEPES/KOH pH 7.6, 0.5 mM CaCl_2_). Imported proteins were separated by SDS-PAGE and analyzed by exposition on X-ray films (Kodak, Perkin Elmer, Rodgau, Germany). In some cases, prior to import chloroplasts were treated with thermolysin (1 mg/ml) for 20 min on ice. The protease treatment was terminated by adding 10 mM EDTA and chloroplasts were washed twice in EDTA containing washing buffer. For import, they were resuspended in import buffer. After import, chloroplasts were treated with 100 μg/ml thermolysin for 20 min on ice, which was also quenched by adding 10 mM EDTA. For competition experiments, overexpressed proteins were added to the import mix in the indicated concentrations. The intensity of radioactive bands were analyzed with the ImageQuant programme (GE Healthcare).

### Protein expression and purification

All recombinant proteins were expressed in *E.coli* BL21 (DE3) pLysS or BL21 (DE3) cells. Cells were grown at 37°C in LB medium in the presence of 100 μg/ml Ampicillin to an OD_600_ of 0.6. Expression was induced by addition of 1 mM isopropyl-1-thio-β-D-galactopyranoside (IPTG), and cells were grown for either 3 h at 37°C or overnight at 12°C. All soluble proteins were purified via their C-terminal His-tag using Ni^2+^-NTA Sepharose (GE Healthcare, Munich, Germany) under native conditions and eluted by increasing the imidazole concentrations. The proteins were concentrated and the buffer exchanged to 50 mM Tris/HCl, pH 8.0, 150 mM NaCl prior to use.

For purification of inclusion bodies (psToc120A), cells were lysed in lysis buffer (50 mM Tris/HCl pH 8.0, 150 mM NaCl, 5 mM β—mercaptoethanol) and centrifuged for 30 min at 14,000 rpm. The resulting pellet was resuspended in detergent buffer (20 mM Tris/HCl pH 7.5, 1% deoxycholic acid, 1% Nonidet P40, 200 mM NaCl, and 10 mM β—mercaptoethanol) and centrifuged for 10 min at 10,000 rpm. The pellet obtained was washed twice with Triton buffer (20 mM Tris/HCl pH 7.5, 0.5% Triton X-100, and 5 mM β—mercaptoethanol) and two times in Tris buffer (50 mM Tris/HCl pH 8.0, 10 mM DTT). The inclusion bodies were finally incubated in buffer A (8 M urea, 50 mM Tris/HCl pH 8.0, 100 mM NaCl, 2 mM β—mercaptoethanol) at RT for 1 h, centrifuged for 10 min at 10,000 rpm and the supernatant was incubated with Ni-sepharose fast flow (GE Healthcare) for 1 h at RT. The Sepharose was washed twice with washing buffer B (8 M urea, 50 mM Tris/HCl pH 8.0, 100 mM NaCl, 40 mM imidazole, 2 mM β—mercaptoethanol) and buffer C (8 M urea, 50 mM Tris/HCl pH 8.0, 1 M NaCl, 40 mM imidazole, 2 mM β—mercaptoethanol). Proteins were eluted by increasing the imidazole concentration to 400 mM. For further purification the protein was eluted from SDS-gels as described in 2.6.

### Generation of antiserum against psToc120A

For immunization of rabbits, inclusion bodies containing psToc120A were purified and separated on 10% SDS-PAGE. The overexpressed protein was excised from the gel and electro-eluted. It was subsequently dialyzed against 50 mM Tris/HCl (pH 7.0), 150 mM NaCl and used to immunize a rabbit, which was sacrificed after five consecutive boosts to obtain polyclonal antiserum.

### Isolation and transient transformation of arabidopsis protoplasts

Mesophyll protoplasts were isolated from leaves of 3 to 4-week-old Arabidopsis plants grown on soil. Leaves were cut into small pieces and incubated in 10 ml enzyme-buffer (1% Cellulase R10, 0.3% Macerozyme R10, 40 mM Mannitol, 20 mM KCl, 20 mM MES pH 5.7, 10 mM CaCl_2_, 0.1% BSA) in the dark for 90 min at 40 rpm. Protoplasts were released by shaking for 1 min at 80 rpm, filtered with a 100 μm Nylon-membrane and centrifuged for 2 min at 100 × g. Protoplasts were resuspended in 500 μl MMg buffer (400 mM Mannitol, 15 mM MgCl_2_, 4 mM MES pH 5.7), separated on a gradient made by 9 ml MSC buffer (10 mM MES, 20 mM MgCl_2_, 1.2% sucrose, pH 5.8) and 2 ml MMg buffer via centrifugation 10 min at 75 × g. Intact protoplasts were washed once in W5 buffer (150 mM NaCl, 125 mM CaCl_2_, 5 mM KCl, 2 mM MES pH 5.7) and resuspended in MMg buffer. 100 μl protoplasts (about 4 × 10^4^ protoplasts) were mixed with 10–50 μg DNA (GFP-fusion constructs) and with 110 μl PEG buffer (40% PEG 4000, Fluka, Germany), 200 mM Mannitol, 100 mM Ca(NO_3_)_2_ and incubated 15 min in dark. Protoplasts were diluted with 500 μl W5 buffer and collected by centrifugation for 2 min at 100 × g. Protoplasts were resuspended in 1 ml W5 buffer and incubate at 25°C overnight in dark. GFP fluorescence was observed with a TCS-SP5 confocal laser scanning microscope (Leica, Wetzlar, Germany).

### Chemical cross-linking

Outer envelope membranes were prepared from pea chloroplasts according to Cline et al. (1981) and incubated with a synthetic peptide representing the N-terminal region of FSD1 (amino acids 1–25) carrying a C-terminal biotin-moiety (JPT Peptide Technologies GmbH, Berlin, Germany) in the presence of 0.1 mM ATP at 4°C for 5 min. After recovery of the membranes by centrifugation they were treated with 5 mM of N-(α-Maleimidoacetoxy) succinimide ester (AMAS, Thermo Fisher Scientific, Schwerte, Germany) for 30 min on ice to initiate cross-linking. Subsequently, the vesicles were solubilized with 1% dodecylmaltoside and incubated with a streptavidin-sepharose matrix. Bound proteins were eluted by addition of Laemmli buffer (60 mM Tris-Cl pH 6.8, 2% SDS, 10% glycerol, 5% β-mercaptoethanol, 0.01% bromophenol blue) and boiling for 3 min. The eluates were separated by 12.5% SDS-PAGE followed by immuno-detection of the biotinylated hybrid protein using VECTASTAIN® ABC system (Biozol, Eching, Germany) or silver staining. The control contained everything except biotinylated peptides and was treated in exactly the same manner. Bands corresponding to specific cross-link products were excised from the gel and sent for analysis via LC-MS/MS. Briefly, the proteins were digested with trypsin in the gel and tryptic peptides were detected by LC-MS/MS. Protein identification was accomplished by a Mascot software assisted database search. Only hits displaying a threshold score of ≥ 60 were analyzed further.

### Peptide array affinity assay

Customized FSD1 peptide arrays were ordered from JPT Peptide technologies. Peptides were synthesized at 5 nmol/spot with acetylated N-termini and covalently bound by C-termini with a polyethylene glycol linker to the cellulose membrane. The recombinant A-domain of psToc120 (psToc120A) was analyzed in the affinity arrays. The peptide array was blocked with 0.3% skim milk in 1X TBS buffer for 1 h and subsequently incubated with 5 μg/ml psToc120A (in 50 mM Tris/HCl (pH 7.0), 150 mM NaCl) overnight at 4°C. The binding of the proteins to the peptides was detected after 3 h incubation with rabbit anti-psToc120A (1:500, 0.3% skim milk in 1X TBS) primary antibody and 1 h incubation with HRP-conjugated anti-rabbit (1:20,000, 0.3% skim milk in 1X TBS) secondary antibody. The membrane was washed three times for 10 min with 0.3% skim milk in 1X TBS after primary and secondary incubation. A negative control was performed by excluding psToc120A from the incubation protocol. The detection was performed with ECL Plus detection reagents. The intensities of the spots were analyzed with ImageQuant TL 8.1 software (GE healthcare, Munich).

## Results

### Sub-cellular analysis of putative “non-canonical” chloroplast proteins

As a first approach to characterize the molecular identity of the of the TOC/TIC-independent transport pathway, a subset of 9 tentative non-canonical chloroplast proteins was selected as baits to “fish” for the potential candidates of the TOC/TIC-independent transport machinery. The main criterion for the selection was based on their robust prediction for the lack of a chloroplast transit peptide (cTP). In order to increase the maximum accuracy of the cTP prediction, nine different prediction algorithms were employed. Only proteins that were predicted to lack a chloroplast transit peptide by at least six out of the nine prediction algorithms were selected (see Supplemental Table 2).

**Figure 1:**
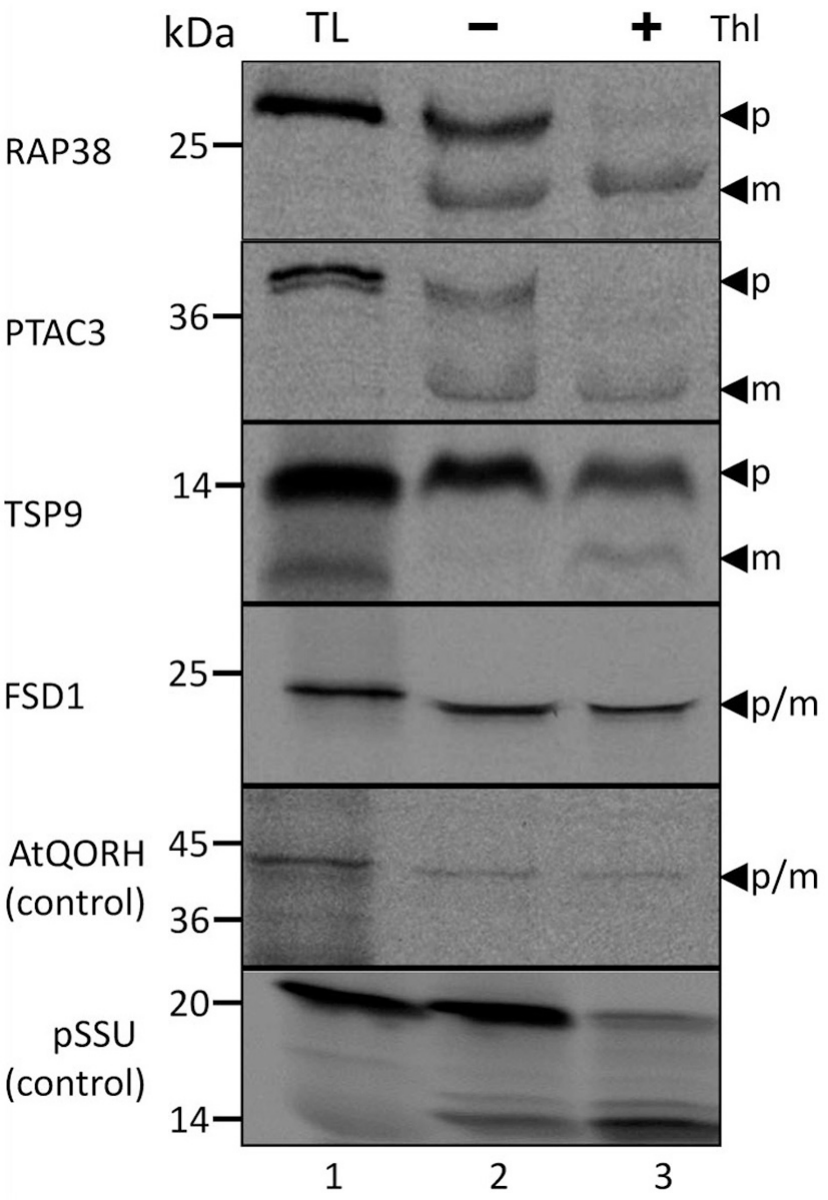
**Import of predicted non-canonical chloroplast proteins**. *In vitro* import assays into pea chloroplasts are shown. TL represents 10% of translation product, Thl (+) indicates thermolysin digestion after the import reaction. The arrowheads indicate precursor (p) and mature (m) proteins. Please note that the mature band of Tsp9 is only visibly present the lane Thl (+), thus it cannot be excluded to have originated from the protease treatment. Representative results from *n* = 3 independent experiments are depicted.

Though all the proteins in the test set have a reported chloroplast localization as well as a computationally predicted lack of transit peptide in previous proteomic studies (Ferro et al., 2003; Kleffmann et al., 2004; Zybailov et al., 2008), the chloroplast localization and processing of these proteins were re-accessed by *in vitro* import assays (Figure 1). In the *in vitro* import assays, detection of the protein prior to thermolysin treatment indicates that the protein has attached to the organelle (Thl −); if the signal persists after thermolysin treatment, it can be inferred that import has occurred (Thl +). Moreover, the presence of an additional smaller band is characteristic for post-import cleavage of the cTP. As control of the TOC/TIC-dependent and TOC/TIC-independent pathways, respectively, the precursor of the small subunit of ribulose-1,5-bisphosphate carboxylase/oxygenase (pSSU) and inner chloroplast envelope quinone-oxidoreductase homolog (AtQORH) were used. Figure 1 depicts the results of import assays for four chosen Arabidopsis proteins which successfully translocated into pea chloroplasts and protease resistant after import. Three proteins were found to be processed to a smaller mature protein that was protease resistant (RAP38, PTAC3, and TSP9). Thus, in deviation from the computational prognosis these proteins do feature a cleavable transit peptide, indicating that the applied algorithms have still to be optimized. The exception was FSD1 (iron dependent superoxide dismutase, At4g25100); similar to the control protein AtQORH, FSD1 did not undergo a proteolytic maturation during import. Since the band visible after FSD1 import ran a little lower in the gel compared to the translation product, we checked the proteomic data (Ferro et al., 2010) for N-terminal peptides. As shown in Supplemental Figure 1 the extreme N-terminus only missing the start methionine was found in the proteomic approach. These observations strongly indicated that FSD1 had been imported without detectable proteolytic cleavage, a result that is consistent with the proteomic studies and the *in silico* analyses. To exclude that FSD1 is intrinsically protease resistant and that the band persistent after thermolysin treatment represents merely surface bound protein, we treated translation products of FSD1 as well as pSSU and AtQORH with thermolysin (Supplemental Figure S2). All proteins proved to be susceptible to thermolysin. Since FSD1 was the only protein in the set that was not processed after import, which indicates the use of a potential alternative translocation mechanism, we further analyzed its import characteristics and used it as a bait to detect potential novel interaction partners at the outer envelope.

### Import characteristics of FSD1 indicate that it uses some general components but shows distinct properties

Successive translocation of precursor proteins across the chloroplast membranes is an energy-dependent process involving the hydrolysis of nucleoside triphosphates at the outer envelope, in the intermembrane space and in the stroma (Pain and Blobel, 1987; Kouranov and Schnell, 1997; Young et al., 1999). Generally, a concentration of nucleoside triphosphates above 100 μM is required for efficient translocation of a standard precursor protein across the envelopes and into the stroma (Theg et al., 1989). To screen for the energetic requirement of FSD1 translocation across the chloroplast envelope membranes, the endogenous nucleoside triphosphates were first depleted from the ^35^S-radiolabeled FSD1 translation products via gel filtration prior to the *in vitro* import assay. Likewise, to minimize the production of endogenous nucleoside triphosphates, chloroplasts were pre-incubated in the dark for 30 min on ice and the subsequent import reactions were carried out in the dark (Flügge and Hinz, 1986; Chen and Li, 1998). Consequently, only the influence of the externally added nucleoside triphosphates on the import characteristics was investigated. As a control for the energy state of stromal-localized proteins during the import reaction, the import of ^35^S-radiolabeled pSSU was also monitored.

**Figure 2:**
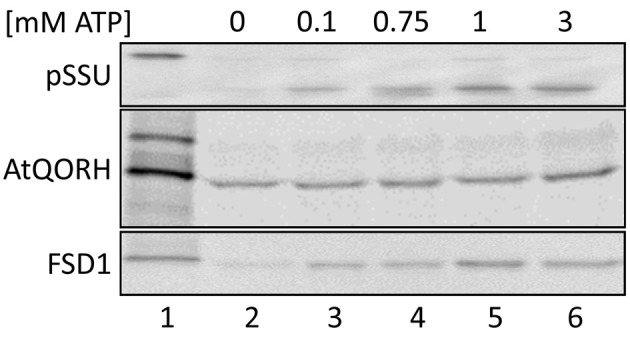
**Energy dependence of FSD1 import**. *In vitro* import assays into pea chloroplasts are depicted. Lane 1 shows 10% of translation product. All samples were treated with thermolysin after import (lanes 2–6). ATP-concentrations in mM are indicated on top of the panel. Representative results from *n* = 3 independent experiments are depicted.

As depicted in Figure 2, import of FSD1 is considerably diminished in the absence of ATP (lane 2), whereas the addition of exogenous ATP clearly resulted in a yield increase (lanes 3–6). Maximal import is reached at 1 mM ATP (100%) whereas no addition gives a yield of ~30% ± 5%. A similar import behavior was observed for pSSU (initial import ~16% ± 2.5%). In both cases a very slight yield decrease in the presence of 3 mM ATP compared to 1 mM ATP can be observed (lanes 5/6), but since this was not a consistent effect in all experiments it seems not to be specific (data not shown). The import of the inner envelope protein AtQORH, on the other hand, was not influenced by the absence of nucleoside triphosphates. Without external ATP the import yield is already higher than 70 ± 13%. Thus, it can be concluded that the import of FSD1 is energy dependent comparable to a general import pathway substrate.

**Figure 3:**
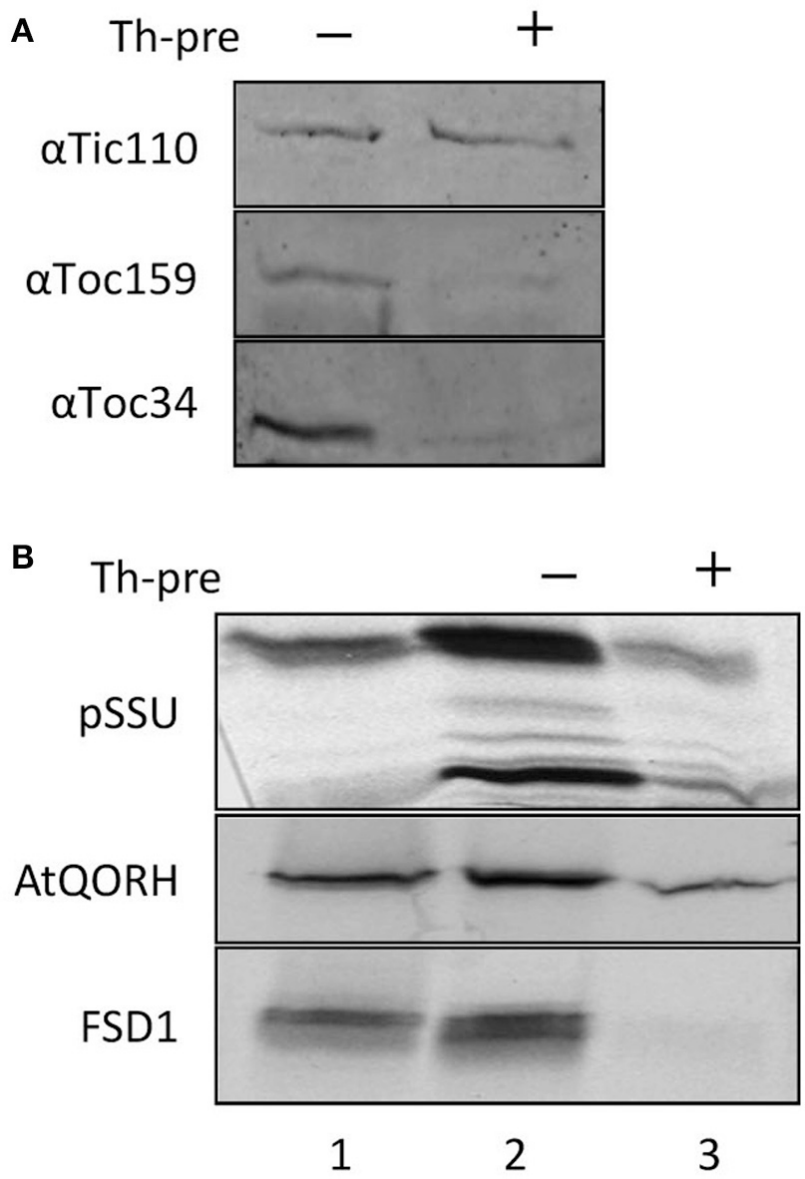
**FSD1 import critically depends on protease sensitive surface receptors. (A)** Immunoblot of chloroplasts used for import experiments before and after thermolysin pre-treatment. The membrane was probed for Tic110, Toc159, and Toc34. Representative results from *n* = 3 independent experiments are depicted. **(B)** *In vitro* import assays into pea chloroplasts are depicted. Lane 1 shows 10% of translation product. Thl-pre (+) indicates thermolysin digestion before the import reaction (lane 3). All samples were treated with thermolysin after import.

Import of canonical precursor proteins into chloroplasts requires protease-sensitive components at the outer envelope membrane (Perry and Keegstra, 1994). To investigate whether this is the case for FSD1, import experiments were performed with purified pea chloroplast after thermolysin pre-treatment (Figure 3). Import reactions of precursor proteins that require intact proteinaceous components should be inhibited after protease treatment. The thermolysin pre-treatment was assessed by immunoblotting, which showed that surface exposed domains of the receptor proteins Toc159 and Toc34 were proteolytically removed whereas the inner envelope protein Tic110 remained intact (Figure 3A). Chloroplasts from the same batch as applied for the immunoblot analysis were used for the import assays. FSD1 and the control proteins AtQORH and pSSU were incubated with chloroplasts corresponding to 15 μg chlorophyll at 25°C for 10–12 min for FSD1 and AtQORH, and 5 min for pSSU (Figure 3B). Protease pre-treatment resulted in a noticeable decrease in binding and import of pSSU. Intriguingly, the translocation of AtQORH was also diminished, though it had been shown that it does not require the main TOC receptor proteins (Miras et al., 2002). It seems that although AtQORH is still imported into protease treated chloroplasts, a protease sensitive component enhances import efficiency. FSD1 import was completely abolished, demonstrating that FSD1 depends on protease sensitive receptors on the chloroplast surface for the initial recognition step and its subsequent translocation into chloroplasts.

**Figure 4:**
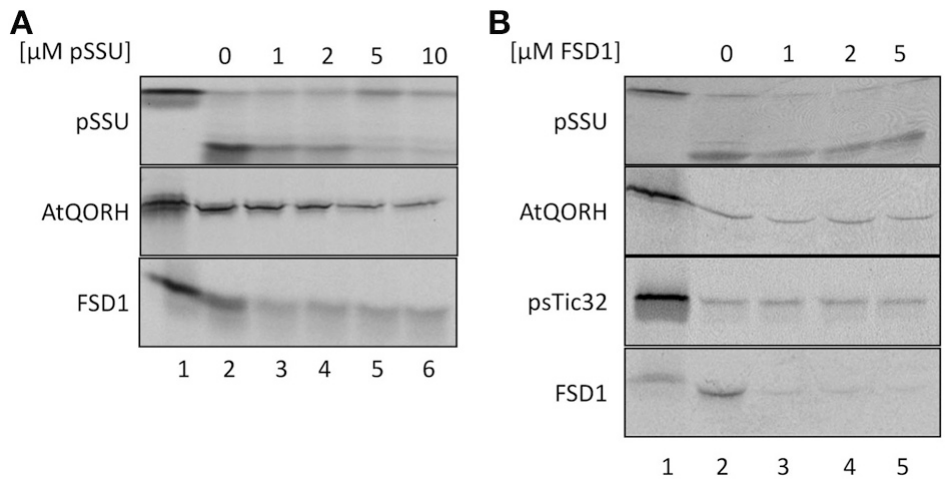
**FSD1 import uses parts of the general import pathway. (A)** *In vitro* import assays into pea chloroplasts are depicted. Lane 1 shows 10% of translation product. Increasing amounts of heterologously expressed pSSU as indicated on top of the panel were added to the import reaction. **(B)** Same as in **(A)** but with FSD1 as competitor. All samples were treated with thermolysin after import. Representative results from *n* = 3 independent experiments are depicted.

To explore if FSD1 makes use of some parts of the general import machinery and/or shares components with AtQORH on its way into the chloroplast, competition experiments with heterologously expressed pSSU and FSD1 were conducted (Figure 4). In a first set of experiments, *in vitro* import experiments were carried out in the presence or absence of different amounts of purified pSSU (Figure 4A). As internal control for the general- and TOC/TIC-independent pathways imports of pSSU and AtQORH were tested in the presence or absence of the unlabeled competitor. The data presented in Figure 4A clearly illustrate that the amount of imported ^35^S-labeled pSSU decreased with increasing concentrations of unlabeled pSSU in a concentration dependent manner by 60–90% ± 6%. The import of ^35^S-radiolabeled AtQORH was only slightly affected in presence of excess pSSU (5 and 10 μM of competitor leads to less than 30 ± 3% of inhibition; lanes 5, 6), when translocation of pSSU itself was already completely abolished (Figure 4A). These observations suggested that AtQORH did not engage the trimeric TOC159/75/34 complex for translocation into the chloroplasts, a result that is consistent with previous work (Miras et al., 2002, 2007). FSD1, on the other hand, confers a clear sensitivity to the presence of the pSSU. At 1 μM concentration of competitor, import of FSD1 is notably diminished (~40 ± 3% inhibition), but in contrast to pSSU the translocation of FSD1 is never completely blocked, even at the highest competitor concentration as judged by the appearance of the mature FSD1 after protease treatment (~50 ± 3%) (Figure 4A). Thus, some translocation components used by FSD1 seem to be blocked by the excess of pSSU, but import could still occur. This could be due to blockage of a single component that both pSSU and FSD1 engage upon import, e.g., Toc75, or of more than one part of the translocon(s) which might be used by both preproteins.

Evidence for the operation of distinct import pathways was previously reported for the import of another inner envelope protein lacking a cleavable transit peptide, psTic32 (Nada and Soll, 2004). Competition experiments were used to address the question of whether FSD1, AtQORH and psTic32 share components of the same import pathway (Figure 4B). For this purpose, heterologously expressed purified FSD1 was added to import reactions containing radiolabeled AtQORH or psTic32. As depicted in Figure 4B, strong competition only occurred in the case of FSD1 itself (~15 ± 3% residual import). From the fact that the import of AtQORH (~60 ± 20%) and psTic32 (75 ± 10%) still occurred in the presence of excess FSD1 it can be concluded that FSD1 translocation does not engage components involved in either psTic32 or AtQORH translocation. The classical precursor protein pSSU shows a slight import inhibition in the presence of recombinant FSD1, which has similar properties as observed in the reciprocal experiment: addition of 1 μM of FSD1 results in an inhibitory effect (80 ± 19% residual import) that is not intensified by higher amounts of competitor. This again implies that pSSU and FSD1 share at least one translocation components on their way into chloroplasts.

### The N-terminus of FSD1 specifically interacts with large outer envelope proteins

**Figure 5:**
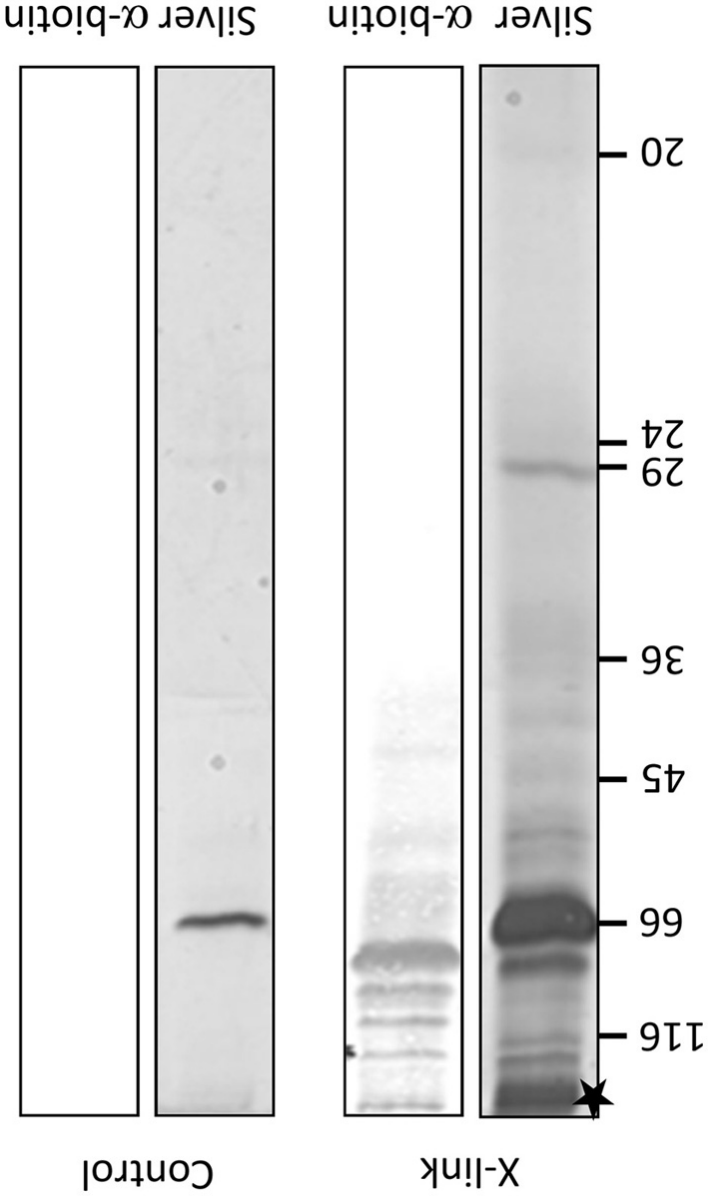
**FSD1 N-terminus interacts with large outer envelope proteins**. A synthetic peptide representing the first 25 AA of FSD1 including a biotin moiety was incubated with outer envelope vesicles, chemically cross-linked and subjected to SDS-PAGE. The samples were analyzed by silver staining and western blot. A band not present in the control was analyzed by mass spectroscopy (indicated by an asterisk).

For most internal chloroplast proteins the N-terminal sequence contains essential targeting information: classical precursor proteins comprise cleavable N-terminal transit peptides, but also Tic32 has its non-cleavable targeting signal in the N-terminus. The only known exception is AtQORH, which is guided by internal sequence information (Miras et al., 2002). Since FSD1 clearly revealed some import characteristics similar to canonical precursor proteins, we decided to address the targeting properties of the extreme N-terminus of FSD1. To this end, we designed a synthetic peptide consisting of amino acids 1–25 including a C-terminal biotin-moiety and applied this to identify possible binding partners of FSD1 at the outer envelope. The peptide was used as bait in a chemical cross-linking approach utilizing right side out outer envelope vesicles (Waegemann et al., 1992) at the energy-independent ‘binding’ stage, reflecting the conditions upon preprotein recognition at the chloroplast surface. The N-terminal FSD1-biotin peptide was used in the presence of 0.1 mM ATP and incubated at 4°C for 5 min. Afterwards, membranes were treated with 5 mM N-(α-Maleimidoacetoxy) succinimide ester (AMAS) for 30 min on ice and then solubilized with 1% dodecylmaltoside. The suspension was incubated with a streptavidin matrix and bound protein complexes were eluted by adding Laemmli buffer. The eluates were separated by 12.5% SDS-PAGE followed by immuno-detection with a α-biotin antibody or silver staining. As depicted in Figure 5, several cross-linked products were observed in the silver stained gel, whereas one prominent band was recognized by the biotin-antibody. No cross-reactivity was observed with an empty streptavidin-sepharose matrix as shown in the control reactions (Figure 5, control). The indicated band (Figure 5, asterisk) was excised from the gel and used for analysis via automated nano-spray LC-MS/MS.

The identified peptides matched 100% with peptides deduced from partial cDNA sequences in the pea database (Franssen et al., 2011) (see Table 1), annotated as psToc120 (Ps200709_Contig058064, Ps200709_Contig051235, Ps200709_Contig017379, Ps200709_Contig017379 and Ps_contig_mira-and-tgicl-ass_9502, Ps_contig_mira-and-tgicl-ass_37108, Ps_singlet_mira-and-tgicl-ass_6441, Ps_singlet_mira-and-tgicl-ass_7910, whereas psToc132 corresponds to Ps200709_Contig020540, Ps200709_Contig020541, Ps200709_Contig051331, Ps200709_Contig060418, Ps200709_Contig060420, Ps200709_Contig062910 and Ps_singlet_mira-and-tgicl-ass_6440, Ps_singlet_mira-and-tgicl-ass_7911.

**Table 1:** **Identification of psToc132 and psToc120 by LC-MS/MS**.

**Matched peptides of psToc132**	**Observed mass**
R.LFVK.E	310.2127
K.FCNFR.R	372.1681
K.DLAYTLR.S	426.2348
K.LQMIRVKFLRLANRL.G	710.3957
K.ATSLGFDMQTVGK.D	685.8334
K.EKIPVSFSGQVTK.D	581.8261
**MATCHED PEPTIDES OF psToc120**	
R.VNYTVSDTQPR.K	640.3153
R.PAGLGSAAPLLEPAAR	745.9187
R.KTEDSSIGEADEYDETR.R	648.9470

Though it has been shown in Arabidopsis that different paralogs of Toc159 assemble into distinct TOC complexes that accept different precursor proteins, no biochemical evidence has been brought forward thus far demonstrating the existence of such a gene family in pea. Therefore, the identification of peptides representing psToc132 and psToc120 from the MS data raised the possibility that a similar multigene family could also be present in pea.

Since the identified peptides were located in the middle of the putative psToc120/psToc132 homologs, we performed 5'-RACE PCRs with degenerated oligonucleotides matching the peptides to isolate N-terminally complete cDNAs from pea. The 5'-RACE PCR amplification for psToc120 produced a product with the size of 1465 bp, whereas the reaction for psToc132 resulted in a product of 1437 bp. The first cDNA clone contained an open reading frame of 1157 bp which encodes for the 391 amino acids, the putative A-domain of psToc120 with a calculated molecular mass of 43.4 KDa (Supplemental Figures 3, 4). Sequence comparison of the deduced amino acid sequence showed 35.5% sequence similarity to atToc120. The nucleotide sequence of the second cDNA clone showed 38.6% sequence similarity to the A-domain of atToc132. This clone, however, lacked a true start methionine, therefore was most likely N-terminally incomplete. In addition, the abundance of the acidic amino acids glutamic acid and aspartic acid as well as the hydroxyl-containing serine and threonine that has been proposed as characteristic features of the A-domain in Arabidopsis Toc159 homologs (Agne et al., 2010; Chang et al., 2012) was also found in both putative A-domain sequences of psToc132 and psToc120. Therefore, both sequences represent genuine A-domains of psToc132 and psToc120 in pea, respectively.

### FSD1 import is inhibited by the presence of psToc120 A-domain

**Figure 6:**
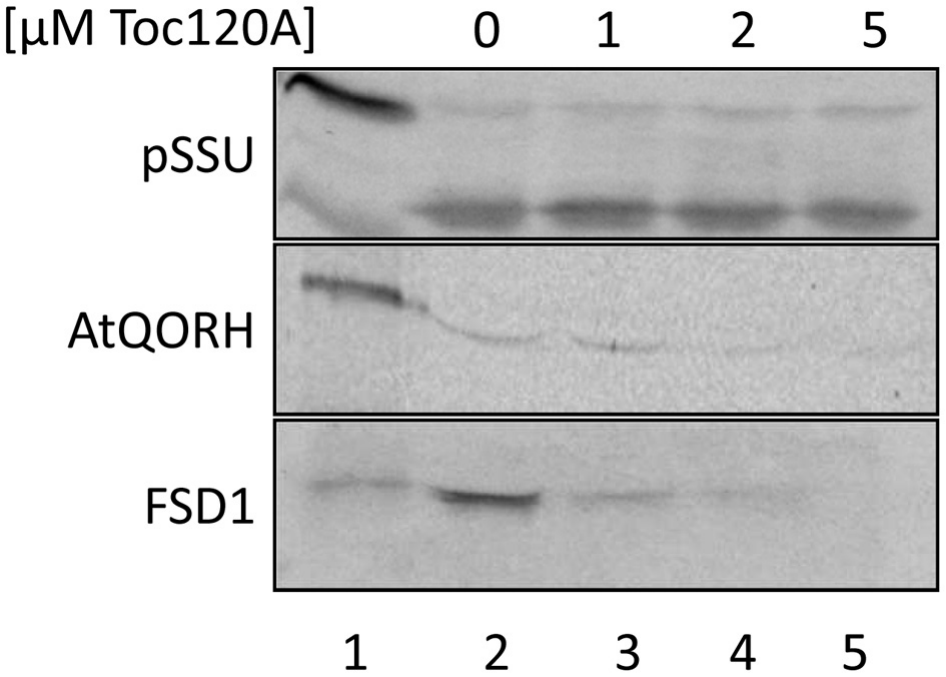
**FSD1 import is inhibited in the presence of psToc120 A-domain**. *In vitro* import assays into pea chloroplasts are depicted. Lane 1 shows 10% of translation product. Increasing concentrations of purified psToc120 A-domain were incubated with *in vitro* translated chloroplast proteins prior to import. All samples were treated with thermolysin after the reaction. Representative results from *n* = 3 independent experiments are depicted.

The coding region for amino acids 1–391 of psToc120, representing the A-domain, was cloned into pET21d with a C-terminal His-tag and heterologously expressed in *E.coli* BL21 (DE3) pLysS. The same approach was initiated for psToc132 A-domain but remained unsuccessful since no heterologous expression could be achieved under various conditions. Thus, we focused on the characterization of psToc120. The purified protein (psToc120A) was used to generate a specific antiserum and as a competitor in import assays. To the latter end, increasing amounts of recombinant psToc120A were added to *in vitro* import assays containing radiolabeled FSD1, AtQORH or pSSU, respectively. A concentration dependent inhibition of FSD1 translocation was observed in the presence of psToc120A (Figure 6, lanes 3–5): at 1 μM psToc120A FSD1 import decreased by ~10 ± 2%, at 2 μM psToc120A by ~45 ± 2% and at 5 μM psToc120A inhibition was already ~70 ± 2%. This most likely indicates that the A-domain of psToc120 binds to FSD1 and thus blocks binding to the intrinsic receptor at the chloroplast surface. A second possibility could be that the heterologously expressed A-domain associates, e.g., with the second receptor Toc34, thus preventing effective translocation of the precursor protein into the chloroplast. In contrast, pSSU import remained unchanged. Intriguingly, a similar inhibitory effect as for FSD1 was observed for AtQORH: at 1 μM psToc120A inhibition was ~20 ± 8%, at 3 μM and 5 μM psToc120A ~50 ± 8%. These data suggest that psToc120 might act as a common receptor for both FSD1 and AtQORH, though AtQORH is still imported to a certain extent after protease pre-treatment in contrast to FSD1 (Figure 3B). This could be due to AtQORH having further protease resistant receptors at the chloroplast surface or a much higher affinity to its import channel, so that it could bypass the receptor.

### The extreme N-terminus of FSD1 is necessary but not sufficient for import, whereas the extreme C-terminus is dispensable

**Figure 7:**
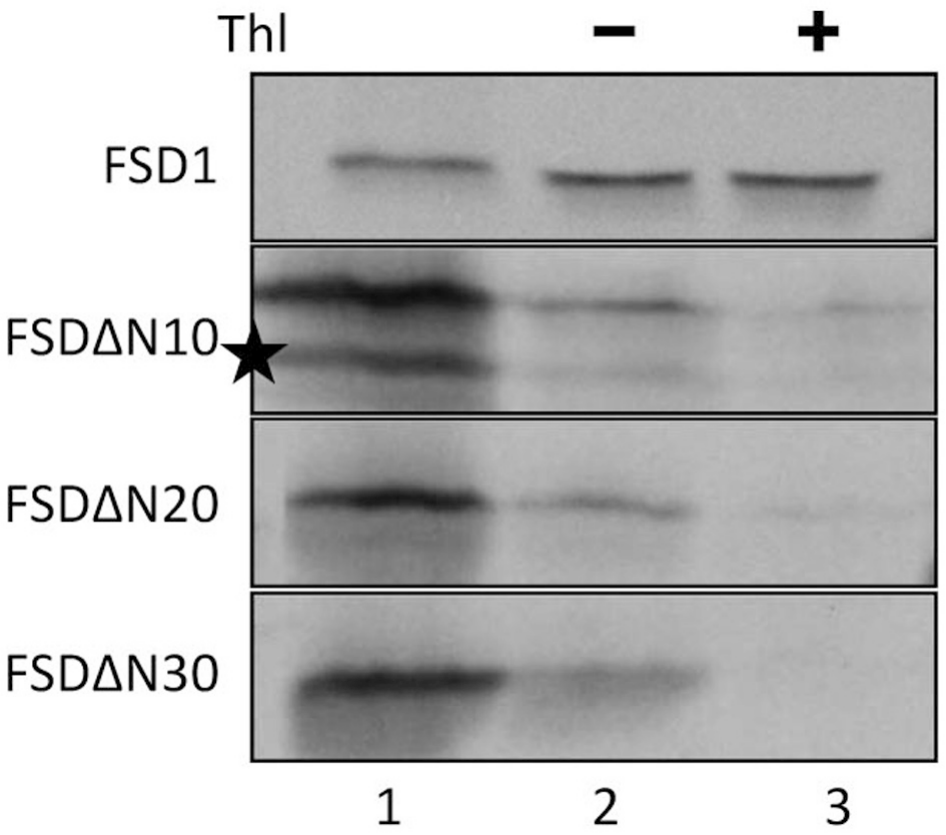
**The extreme N-terminus of FSD1 is indispensable for import**. *In vitro* import assays into pea chloroplasts are shown. Lane 1 represents 10% of translation product, Thl (+) indicates thermolysin digestion after the import reaction (lane 3). Please note the second band in the translation product of FSD1DN (indicated by an asterisk) which might derive from a downstream methionine recognized in the reticulocyte lysate as an alternative start. Representative results from *n* = 3 independent experiments are depicted.

**Figure 8:**
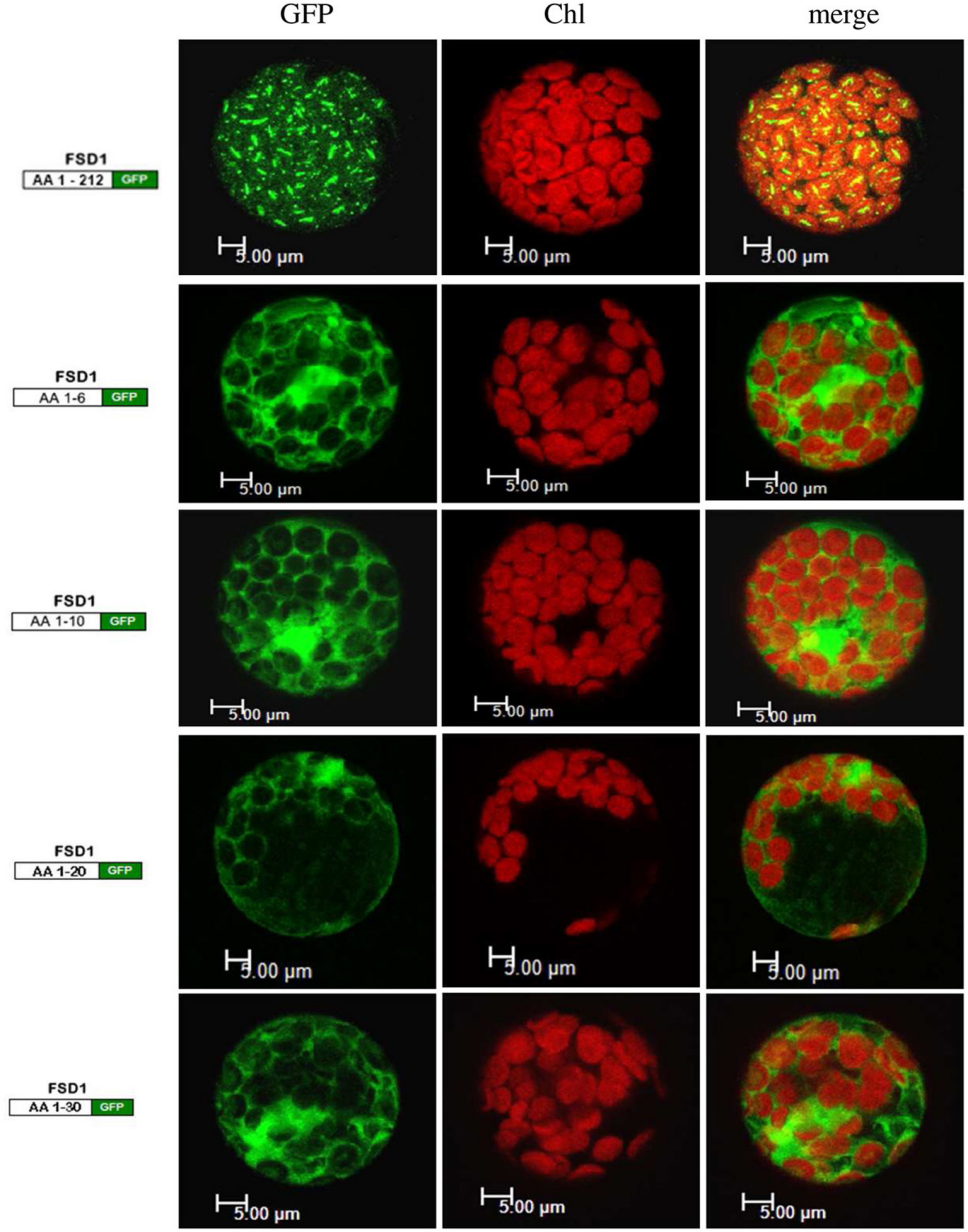
**The extreme N-terminus of FSD1 is not sufficient for guiding a reporter into chloroplasts**. Arabidopsis mesophyll protoplasts were transiently transformed with C-terminal GFP fusions to either full-length FSD1 or the indicated number of N-terminal amino acids. Maximum intensity signals from confocal images are shown for GFP fluorescence (GFP), chlorophyll autofluorescence (chl) and an overlay of both (merge). The used constructs are depicted on the left. *AA*, amino acids; bar, 5 μm.

**Figure 9:**
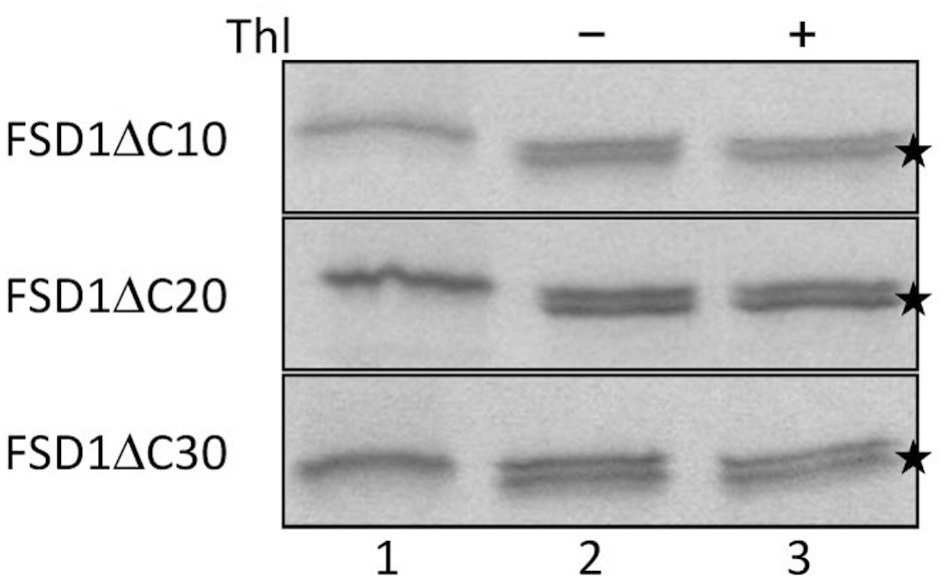
**The extreme C-terminus of FSD1 is not necessary for import**. *In vitro* import assays into pea chloroplasts are shown. Lane 1 represents 10% of translation product, Thl (+) indicates thermolysin digestion after the import reaction (lane 3). Asterisks indicate lower bands probably arising from post-translational modifications such as processing of the first methionine. Representative results from *n* = 3 independent experiments are depicted.

Since the N-terminal 25 amino acids of FSD1 were found in a complex with a TOC receptor protein and primary amino acid sequence comparison between FSD1 and its prokaryotic homologs revealed that the N-terminal region (eight amino acids in at FSD1, 40 amino acids in osFSD1 and 32 amino acids in the algal FSD1) is found exclusively in plant FSD1 proteins (Supplemental Figure 5) we strongly suspected that the N-terminus of the plant FSD1 proteins could be required for specific chloroplast targeting. For further examination of the domains or regions mandatory for plastid localization of the FSD1 protein, several truncated versions of the FSD1 protein in which 10–30 amino acids were progressively removed from the N-terminus were generated. Full-length FSD1 and the deletion mutants were subsequently synthesized *in vitro* and used for import studies. The shortened proteins showed a remarkable reduction in the extent of chloroplast binding. With the exception of the full-length FSD1 all truncated proteins remained protease accessible, demonstrating that no productive translocation into chloroplasts had occurred. Deletion of the first 10 amino acids already abolished import (Figure 7, FSD1ΔN10) indicating that the N-terminus of FSD1 constitutes an indispensable part in signal recognition and targeting to the chloroplast. The same observation was made for FSD1ΔN20 and FSD1ΔN30 (Figure 7). To investigate whether the first 10 amino acids at the N-terminus of FSD1 are not only necessary but sufficient to drive translocation of FSD1 to plastids *in vivo*, the localization of partial or full-length FSD1-GFP fusion constructs were monitored via transient transformation of Arabidopsis mesophyll protoplasts. Intriguingly, while the 10 amino acids at the N-terminus of FSD1 are essential for plastid recognition, they were not sufficient to direct the GFP construct into plastids (Figure 8). For all chimeric constructs except the full length FSD1-GFP a cytosolic localization of the fluorescent signal was observed. This demonstrated that the N-terminal part of FSD1 (30 first residues) is not sufficient for plastid localization of a reporter protein. To establish if the C-terminus also contains important targeting information, we generated C-terminal deletion constructs where 10, 20, or 30 amino acids were missing, FSD1ΔC10, FSD1ΔC20, and FSD1ΔC30, respectively (Figure 9). None of these proteins were affected in translocation efficiency, indicating that the extreme C-terminus of FSD1 is not necessary for targeting or import into chloroplasts. Please note that upon import of the C-terminal deletion constructs two protease resistant bands appeared (indicated by asterisks), which could be due to unspecific proteolytic cleavage of the non-native substrate.

### psToc120A has specific recognitions motifs in FSD1

**Figure 10:**
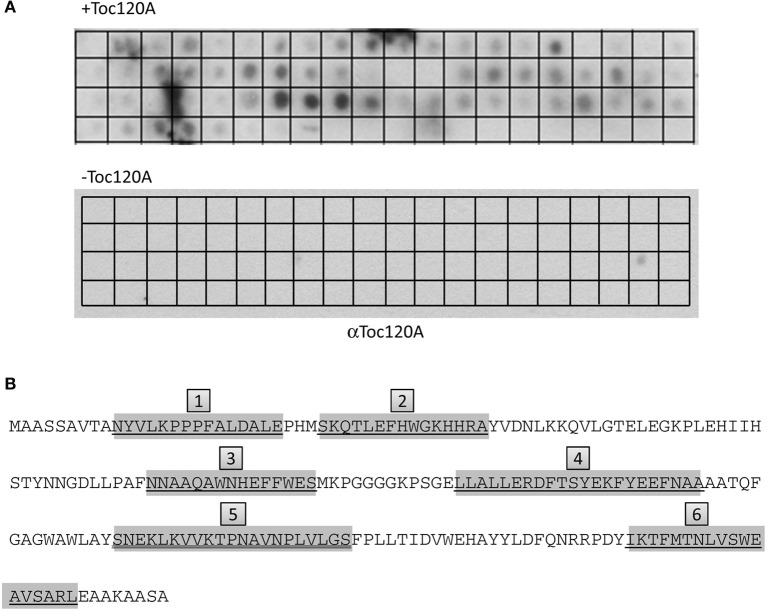
**psToc120A domain has specific recognition motifs in FSD1**. PepSpot assays were designed and spotted onto filter paper where each peptide represents consecutively 15 AA with an overlap of 12 AA. In total, 67 peptides were spotted; the last 13 spots of the membrane are devoid of peptide. **(A)** Heterologously expressed psToc120A was incubated with the filters and detected by immunoblotting with a specific antibody against psToc120A. **(B)** Prominently bound peptides are marked in gray in the FSD1 sequence and the deducted binding regions are numbered form one to six in gray boxes.

The results indicated that the N-terminal region of FSD1 is indispensable for targeting, but not sufficient to drive translocation of the fusion protein into chloroplasts (Figures 7, 8), whereas the extreme C-terminus is not necessary for import (Figure 9). This implies that other regions of FSD1 contain additional sequence information that is vital for receptor recognition and/or for the process of protein import. In order to identify the regions in FSD1 that interact with psToc120, a PepSpot assay was designed. The FSD1 peptides spotted on the membrane were 15 amino acids in length, with each subsequent peptide on the array overlapping by 12 amino acids with the previous one. In total, 67 peptides covered the full length sequence of FSD1. This peptide array was incubated with recombinant psToc120A and the binding specificity of the receptor was detected with specific primary and HRP-conjugated secondary antibodies. As revealed in the peptide scan analysis, psToc120 demonstrated a high relative binding specificity across the array (Figure 10). A negative control that was performed in the absence of recombinant psToc120A showed no unspecific binding of the antibodies to the peptides. The psToc120 receptor interacted with several consecutive stretches of peptides that are distributed across the entire sequence of FSD1 (Figure 10B, motifs one to six). The amino acid sequences of the psToc120-binding peptides and the minimal binding motif present in each of the peptides are depicted in Figure 10B. Notably, the identified binding motif of 10-VTANYVLKPPPFALDALE-24 matches the sequence of the N-terminal FSD1-biotin hybrid protein used in the cross-linking assay (see above). This short peptide sequence is most probably indispensable in the initial targeting and recognition of FSD1 by the psToc120 receptor at the chloroplast surface while binding motifs at the C-terminus might be involved in conferring specificity to FSD1 targeting and its subsequent translocation into the chloroplast.

**Figure 11:**
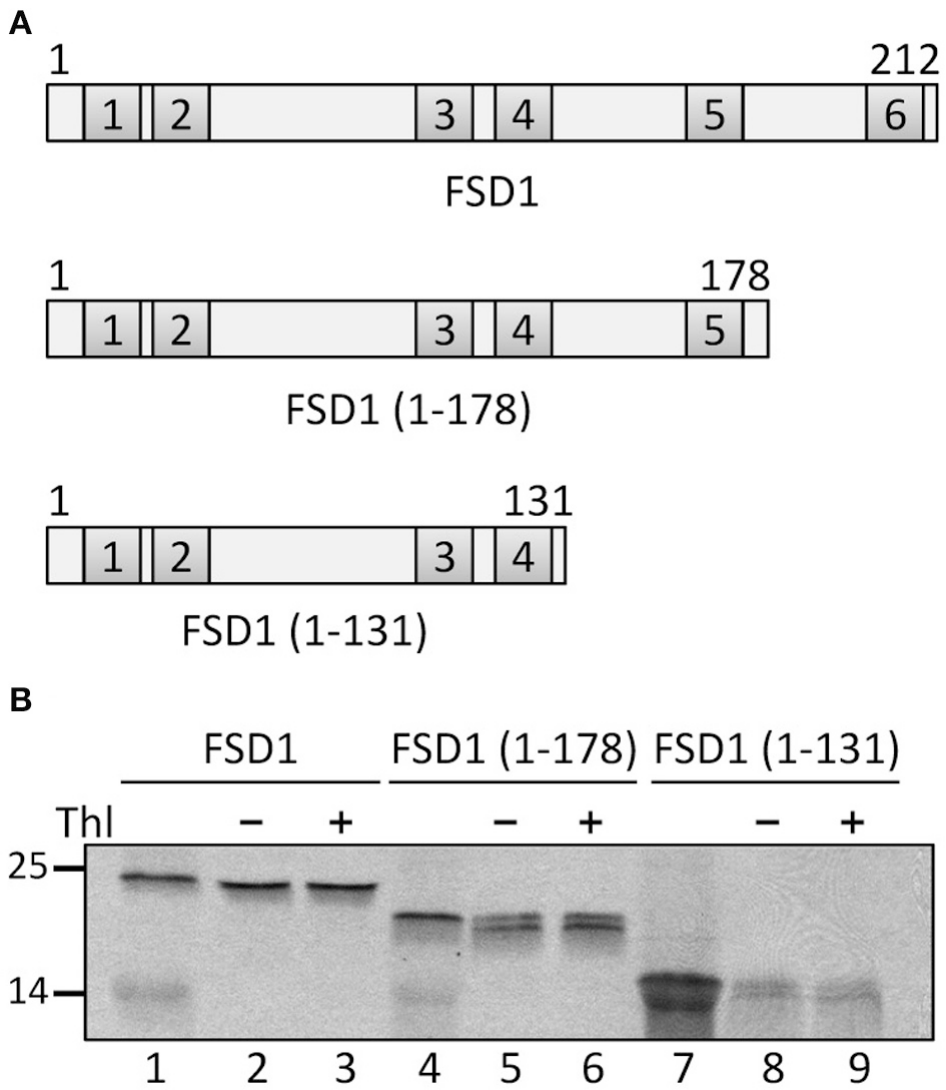
**The fifth recognition motif in the C-proximal end of FSD1 import is essential for binding and import. (A)** Schematic representation of the constructs used for import in **(B). (B)** *In vitro* import assays of C-terminal truncations are depicted. Lane 1 shows 10% of translation product. Thl (+) indicates thermolysin digestion after the import reaction (lanes 3, 6, 9). Numbers on the left indicate size markers in kDa. Representative results from *n* = 3 independent experiments are depicted.

To further confirm the results from the PepSpot assay we generated several truncations of FSD1 for *in vitro* import experiments. These were lacking either only recognition motif six (amino acids 1–178 were still present) or motifs five and six (amino acids 1–131 present), respectively (Figure 11A). Import assays of these proteins revealed that deletion of motif six does not diminish import, which is well in line with the results from the C-terminal deletions displayed in Figure 9. In contrast, after import of an FSD1 construct lacking motif five only weak protease resistant bands could be detected (Figure 11B), demonstrating an obvious inhibition of translocation efficiency. This corresponds perfectly to the intensity observed in the PepSpot assay where binding was strongest to the region defined as motif five. These observations together with the previous data from the *in vitro* interactions studies with the recombinant psToc120A receptor strongly point to a *bona fide* interaction between psToc120 and FSD1 in specific regions of the substrate protein.

## Discussion

The general import pathway is the route taken by the majority of chloroplast proteins. During recent years it turned out, however, that several chloroplast residents travel a different path to reach their destination. Not much is known about these alternative import ways, and only very few substrates have been analyzed so far. To identify substrates for a to date elusive alternative import pathway, we made use of published proteomic studies (Zybailov et al., 2008) from which we chose several proteins with confirmed stromal localization and a strong prediction against the presence of a classical cleavable transit peptide. Nine of these proteins were analyzed with regard to their *in vitro* import behavior and four were found to be amenable to this assay (Figure 1, Supplemental Table 1). To our great surprise, three of the four proteins proved to be processed upon translocation into chloroplasts, which was in stark contrast to the computational predictions. Though we did not investigate the import characteristics of these precursor proteins any further, it seems quite likely that they employ the general import pathway. This clearly shows that the algorithms applied for transit peptide prediction are not yet completely reliable and need to be optimized, which will be a challenging task for the future.

We could, however, identify one protein which was not cleaved upon import and thus constituted a promising substrate for our endeavor. We proceeded to appraise the import characteristics of FSD1 with regard to targeting information contained within the mature sequence, energy dependence and the engagement of known translocation components. It turned out that FSD1 shares some features with substrates of the general import pathway, such as the necessity of the extreme N-terminus for targeting and import (Figures 7, 8), the need for protease-sensitive receptors at the plastid surface (Figure 3) as well as the ATP-dependence of translocation (Figure 2). Though the exact minimal ATP concentration necessary for translocation varies between the different preproteins, import of both pSSU and FSD1 is clearly stimulated at concentrations higher than 0.1 mM ATP. Furthermore, import of FSD1 greatly decreased in the presence of recombinantly expressed pSSU, which travels via TOC/TIC into chloroplasts (Figure 4A). All this infers that FSD1 might engage at least one component of the general import pathway. When we tested for constituents shared with Tic32, another non-canonical chloroplast protein which import mechanism is still elusive, we found that these two proteins obviously travel independent routes, since an excess of FSD1 did not hamper Tic32 translocation (Figure 4B). The control protein AtQORH that was used for a partially characterized non-canonical import pathway behaved differently from FSD1 in almost all assays. This suggests that with FSD1 we discovered a third class of substrate protein, which uses Toc120 and most likely other Toc components such as Toc75 (in fact, we also identified peptides from Toc75 in the excised band from the cross-linking approach depicted in Figure 5), but represents the only preprotein without a cleavable transit peptide identified to date.

To pinpoint specific components of the translocon responsible for the passage of FSD1 into chloroplasts, we applied a chemical cross-linking approach using a synthetic peptide which represented the first 25 amino acids from FSD1 and carried a C-terminal biotin-moiety. This enabled us to detect cross-linked products by using the biotin-streptavidin system. Since the peptide was too short to be fully translocated we opted for focusing on the binding events at the outer envelope membrane. To this end, the peptide was bound to isolated outer envelope vesicles, cross-linking was initiated and the complexes containing the bound peptide finally pulled out by incubation with a streptavidin matrix (Figure 5). A band that appeared exclusively in the cross-linked sample was excised from the gel and sent for MS analysis. Thorough scrutinizing of the resulting peptide masses revealed that the FSD1 peptide bound to two proteins with similarities to the A-domains atToc132 and atToc120, respectively. These proteins belong to the family of atToc159 receptor GTPases and have been shown to build complexes different from atToc159/atToc33/atToc75. Originally, it was speculated that atToc159 is responsible for binding only precursor proteins involved in photosynthetic processes, whereas atToc132/Toc120 are more involved in accepting proteins fulfilling house-keeping functions (Kubis et al., 2004). Upon sequence alignment of the respective A-domains, it turned out that the highest sequence variability between Toc159 paralogs lies within these acidic regions, whereas the G- and M-domains are quite conserved (Ivanova et al., 2004). From swapping A-domains between the different atToc159 isoforms *in planta* it was concluded that receptor specificity is achieved by the respective A-domains (Inoue et al., 2010). This hypothesis was, however, questioned by a recent proteomic study which analyzed the proteome of the *ppi2* mutant plants lacking atToc159 (Bischof et al., 2011). Many proteins involved in photosynthesis have been found to be present in the mutant plastids, clearly implying that import of these pre-proteins does not exclusively rely on atToc159. At least in the mutant plants the other paralogs can complement for the loss of atToc159. How the distinct receptor components distinguish their substrates *in vivo* remains to be elucidated.

**Figure 12:**
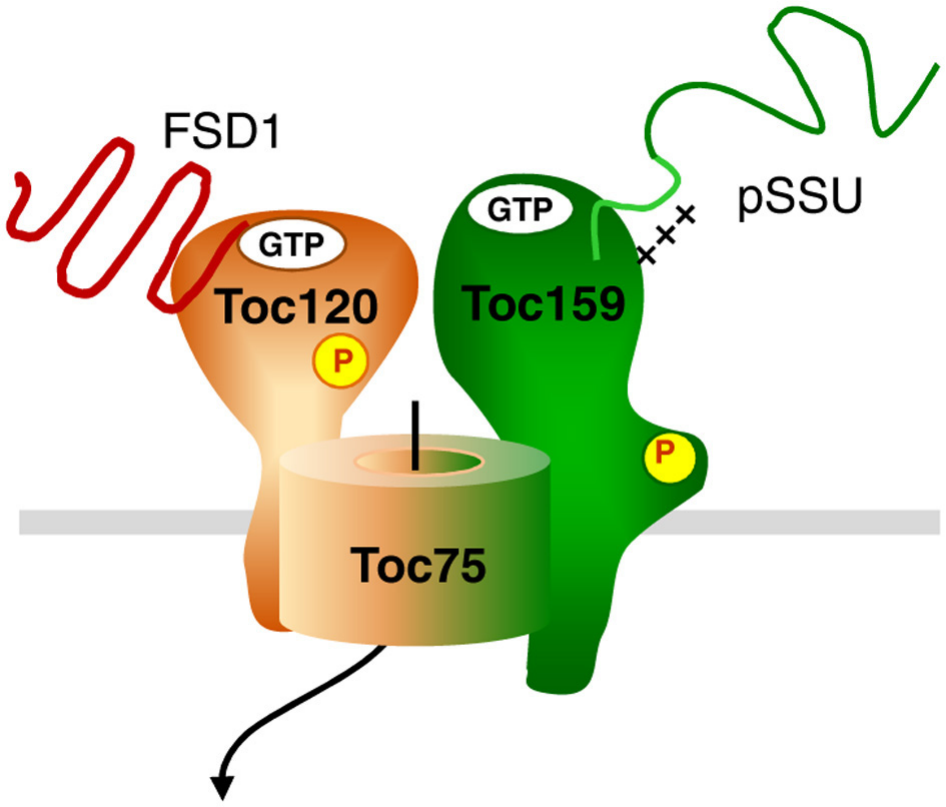
**Hypothetical model for dynamic TOC complexes**. The general import pathway comprises Toc159, Toc34, and Toc75 as core components. The hypothetical translocon responsible for FSD1 import consists of Toc120, most likely Toc75 and unknown component(s). Green color indicates the pathway taken by canonical substrates, whereas orange signifies an alternative translocon. A mix of both colors indicates participation in both translocation machineries. Thus, Toc75 as the common channel can form a complex with both Toc120 and Toc159, but not necessarily at the same time.

FSD1 clearly represents a protein with photosynthesis related function, since the scavenging of reactive oxygen species is highly relevant during active photosynthesis. Thus, one might have expected to find it prominently bound to psToc159. The fact that we found it associated with the newly identified ortholog of atToc120, psToc120, implies that the import pathway which is engaged by a protein might rather depend on intrinsic sequence properties than on its final function within plastids. Concerning the composition of the translocon responsible for FSD1 import other than Toc120, we can only speculate. The fact that we also found peptides from Toc75 argues for FSD1 using the Toc75 import channel. In Arabidopsis Toc120 and Toc132 associate with Toc75 and Toc33/34; this results in the existence of several distinct complexes with the one common element being the channel Toc75. Thus, we hypothesize that FSD1 is specifically recognized by Toc120 (and maybe Toc132) and then engages Toc75. This is exemplarily represented in the model in Figure 12. But at this point it is just a hypothesis which awaits confirmation. Another scenario that could be envisioned is that distinct Toc complexes exist in pea - as has been shown in Arabidopsis—that consist of different combinations of psToc159, −132, and −120 with Toc34. These distinct Toc complexes with different Toc GTPase receptors could have different, but overlapping, substrate specificities accounting for the partial competition of FSD1 for pSSU import. This would be in line with the hypothesized situation in other systems that have already been shown to have multiple Toc159 isoforms.

When we aimed to determine the regions in FSD1 responsible for binding to psToc120 by peptide array analysis, we found that indeed specific areas of the protein bind more strongly to the receptor than others (Figure 10). The reliability of the array was demonstrated by the fact that we detected the N-terminal peptide used for cross-linking among the most strongly bound regions. From that array, we defined six regions within FSD1 which seemed important for binding to the A-domain. They are evenly distributed across the protein including the N- and C-termini. To confirm these data we generated C-terminal truncations to be applied in import assays, which in fact corroborated the regions essential for binding and import of FSD1 (Figure 11). While the extreme C-terminus itself is not important (Figure 9), the C-proximal region five, which is most strongly labeled in the peptide array, proved to be indispensable. This is in line with data reported by Lee et al. (2006), Lee et al. (2009) that even in pSSU, the classical canonical precursor protein, sequence information within the mature part of the protein is required for efficient translocation.

## Author contributions

WaiLing Chang conducted experiments and was involved in drafting of the manuscript. Jürgen Soll and Bettina Bölter designed the work and wrote the manuscript. All authors approved the final version.

### Conflict of interest statement

The authors declare that the research was conducted in the absence of any commercial or financial relationships that could be construed as a potential conflict of interest.
