# Supplementary material for: A new member of the psToc159 family contributes to distinct protein targeting pathways in pea chloroplasts
Source: Front Plant Sci. 2014 May 28;5:239. doi: 10.3389/fpls.2014.00239 (PMC4036074; doi:10.3389/fpls.2014.00239)
Supplement: Supplementary file 1 [file DataSheet1.DOCX]

| **Name** | **Sequence** |
| --- | --- |
| FSD1 | CATGGAATTCATGGCTGCTTCAAGTGCT |
| FSD1∆N10 | CATGGAATTCATGGTCCTCAAGCCACCTCCA |
| FSD1∆N20 | CATGGAATTCATGGCTTTGGAGCCGCATATG |
| FSD1∆N30 | CATGGAATTCATGCTGGAGTTTCACTGGGGA |
| FSD1∆C10 | CATGGGATCCTTAGGCACTTACAGCTTCCCAAG |
| FSD1∆C20 | CATGGGATCCTTAGGTCATGAATGTCTTTATGTAATC |
| FSD1∆C30 | CATGGGATCCTTATCGGTTCTGGAAGTCAAGG |
| FSD1 | CATGGGATCCTTAAGCAGAAGCAGCCTTGGC |
| FSD1.SpeI | CATGACTAGTATGGCTGCTTCAAGTGCTGTC |
| FSD1_.SalI | CATGGTCGACGAGCAGAAGCAGCCTTGGCGGC |
| FSD1(1-6).SpeI | CTAGTATGGCTGCTTCAAGTGCTCG |
| FSD1(1-6).SalI | TCGACGAGCACTTGAAGCAGCCATA |
| FSD1(1-10).SpeI | CTAGTATGGCTGCTTCAAGTGCTGTCACCGCAAACCG |
| FSD1(1-10).SalI | TCGACGGTTTGCGGTGACAGCACTTGAAGCAGCCATA |
| FSD1 (1-20).SpeI | CTAGTATGGCTGCTTCAAGTGCTGTCACCGCAAACTACGTCCTCAAGCCAC CTCCATTCGCACTGCG |
| FSD1 (1-20).SalI | TCGACGCAGTGCGAATGGAGGTGGCTTGAGGACGTAGTTTGCGGGACAGCACTTGAAGCAGCCATC |
| FSD1 (1-30).SpeI | CATGACTAGTATGGCTGCTTCAAGTGCTGTC |
| FSD1 (1-30).SalI | CATGGTCGACGTTGTTTGCTCATATGCG |
| FSD1.NcoI | CATGCCATGGATGGCTGCTTCAAG |
| FSD1.BamHI | CATGGGATCCTTAAGCAGAAGCAGCC |
| GSP1(TOC120) | GCTGCTGCACCACCCGGGCAGCCGGTTC |
| NGSP1(TOC120) | CAAGAGGGGTGCTAGCAGCAACAGATGA |
| GSP1(TOC132) | CTA CCCTGGGAGCAGGTTCCAACAAAGA |
| NGSP1(TOC132) | AGGAGTGCTAGTAGCAACAGAATGTCCAGATGAG |
| PsToc120A.EcoRI | CATGGAATTCATGGATAATGGTGGGTATGATGAG |
| PsToc120A:HindIII | CATGAAGCTTCTGCTGCACCACCCGGGC |
