# Supplementary material for: A new member of the psToc159 family contributes to distinct protein targeting pathways in pea chloroplasts
Source: Front Plant Sci. 2014 May 28;5:239. doi: 10.3389/fpls.2014.00239 (PMC4036074; doi:10.3389/fpls.2014.00239)
Supplement: Supplementary file 2 [file DataSheet2.DOCX]

| **AGI Code** | **Description** | **Annotation** | **Non-cTP prediction**** | **In Vitro Import** |
| --- | --- | --- | --- | --- |
| At4g25100 | Iron-superoxide dismutase 1 | FSD1 | 7 | + |
| At3g47070 | Thylakoid phosphoprotein | TSP9 | 9 | + |
| At2g05620 | Protein of unknown function, essential for photoprotection | PGR5 | 7 | + |
| At1g09340 | Protein of controversially discussed function | Rap38 | 6 | + |
| At4g13010 | Quinone – oxidoreductase | AtQORH | 7 | + |
| At4g20010 | Plastid transcriptionally active 3 | PTAC3 | 8 | + |
| At5g53580 | Putative aldo/keto reductase family | AldKet | 6 | + |
| At4g22930 | Dihydroorotase | PYR4 | 7 | + |
| At4g31050 | Putative lipoate-protein ligase B | Lip2 | 8 | (+) |
| At1g35720 | Stress-responsive calcium-dependent membrane- | AtAnnAt1 | 9 | - |

**Supplemental Table 2: Experimentally determined sub-cellular localization of the putative non-canonical chloroplast proteins**

** Number of algorithms that predict a non-cp location, namely absence of a cTP. In all, nine different predictors

were employed: TargetP (www.cbs.dtu.dk/services/TargetP/‎), Predotar (https://urgi.versailles.inra.fr/Tools/Predotar), ChloroP (www.cbs.dtu.dk/services/ChloroP/), Wolfpsort (wolfpsort.seq.cbrc.jp/‎), iPSORT (ipsort.hgc.jp/‎), (PSORT psort.hgc.jp/‎); PCLR (www.andrewschein.com/cgi-bin/pclr/pclr.cgi‎), BaCelLo (gpcr.biocomp.unibo.it/bacello/‎), ProteinProwler (http://www.mybiosoftware.com/protein-sequence-analysis/8441).

All proteins have been tested for chloroplast localization by in vitro import assays. Those found to be imported are marked by “+”, proteins that did not import are indicated by “-“. Grey shading indicates the proteins depicted in Fig. 1.
