# Supplementary material for: A new member of the psToc159 family contributes to distinct protein targeting pathways in pea chloroplasts
Source: Front Plant Sci. 2014 May 28;5:239. doi: 10.3389/fpls.2014.00239 (PMC4036074; doi:10.3389/fpls.2014.00239)
Supplement: Supplementary file 3 [file Presentation1.PDF]

MAASSAVTANYVLKPPPFALDALEPHMSKQTLEFHWGKHHRAYVDNLKKQVLGTELEGKPLEHIIHST
YNNGDLLPAFNNAAQAWNHEFFWESMKPGGGGKPSGELLALLERDFTSYEKFYEEFNAAAATQFGAGW
AWLAYSNEKLKVVKTPNAVNPLVLGSFPLLTIDVWEHAYYLDFQNRRPDYIKTFMTNLVSWEAVSARL
EAAKAASA

Supplemental Figure 1

SUPPLEMENTAL FIGURE 1 | FSD1 Peptides identified in Ferro et al., 2009. Peptides identified in their proteomic approach (Ferro et al., 2009, supplemental table 7) were matched of the FSD1 sequence and represented in red.
