# Supplementary material for: A new member of the psToc159 family contributes to distinct protein targeting pathways in pea chloroplasts
Source: Front Plant Sci. 2014 May 28;5:239. doi: 10.3389/fpls.2014.00239 (PMC4036074; doi:10.3389/fpls.2014.00239)
Supplement: Supplementary file 4 [file Presentation2.PDF]

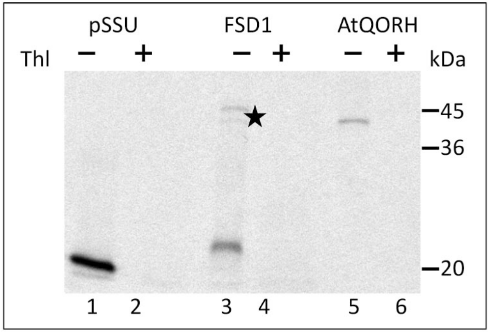

Supplemental Figure 2

SUPPLEMENTAL FIGURE 2 | Protease sensitivity assay. Translation products of pSSU (lane 1), FSD1 (lane 3) and AtQORH (lane 4) were treated or not treated with thermolysin. The asterisk indicates a high molecular weight band, which is probably due to aggregation.
