# Supplementary material for: A new member of the psToc159 family contributes to distinct protein targeting pathways in pea chloroplasts
Source: Front Plant Sci. 2014 May 28;5:239. doi: 10.3389/fpls.2014.00239 (PMC4036074; doi:10.3389/fpls.2014.00239)
Supplement: Supplementary file 5 [file Presentation3.PDF]

```
AACGCAGAGTACGCGGGGAACCAAGACAGTGACAGAAACAGCTGTTCTTCTCTGCATAAACAGGATTTTATAAGGACTTGGAGATTTGTGATGGATAATGGTGGGT :  106
 N A E Y A G N Q D S D R N S C S S L H K Q D F I R T W R F V M D N G G

ATGATGAGGGGGAGAGGAAGAGGGTGGATGTTGGGGTGTCAAATGAGAGTATAGGAGGGTCTTACGAGGAGTTGAAGAGTTCTGAAGGAGATGAGGTTTTTGAGGA :  212
 Y D E G E R K R V D V G V S N E S I G G S Y E E L K S S E G D E V F E

GGCGACTGACCCGTTAAAGGATTTCAATGATCGGGGTGATGCTGTTGCTGCTACTGTTAGTGTATTGCCTTCAGATTTGGTTGAAGAAATTCAGGATGATGATGCC :  318
E A T D P L K D F N D R G D A V A A T V S V L P S D L V E E I Q D D D A

GAAGAACTTGATAGTTTTCTAGAGGCAATTGGGGTTGGTGATGGGCGTGTCAAAGTGTCGGAGGATGAAGAAGAAGTTGAAGTGTTTGACTTTGTGAATGGATTCT :  424
 E E L D S F L E A I G V G D G R V K V S E D E E E V E V F D F V N G F

CTGGTTTATCCCGTGAGAGATTTGAAAACGAGGATGTTGAATACGTTACTCCCAAACAAAATGGTGGTATACTTTTCGAAAATGGGAGCACGGACAAGGTGGATTA :  530
 S G L S R E R F E N E D V E Y V T P K Q N G G I L F E N G S T D K V D

TGATGTCGATGAGTTTCATACGTATTCTGGATCCAATGAGGAGATGAGAAACCAGGGTGCGGAGGACAAGGTGGATTATGATGCCGATGAGTTTCATACGTATTCT :  636
YD V D E F H T Y S G S N E E M R N Q G E D K V D Y D A D E F H T Y S

GGATCCAATGAGGAGATGAGAAACCAGGGTGCGGAGGCTGAGGATTTGAAAGAGGGTGGTTTGGATACAGAACTCCGAGATGATAAAATAATAGAGGAACAATGTA :  742
 G S N E E M R N Q G A E A E D L K E G G L D T E L R D D K I I E E Q C

ATGCTTCGGGTGGCCCTTATAGTGATATTCAAGATGATAAATTGCATATACATTCAGCTCGCGGAGGTTTAGAAATGGAGGGTGAAACCCTTGGCTCTGATGTAGT :  848
 N A S G G P Y S D I Q D D K L H I H S A R G G L E M E G E T L G S D V

CCATGAGGATAGAAATGGCGAAGAAATTGGAATATCTGATTGTCAGAGTATTGAATGCAAAGATTATAGTAATGATGAAGATAAAGGTGCTAGTGCTGAAACAGAT :  954
 V H E D R N G E E I G I S D C Q S I E C K D Y S N D E D K G A S A E T D

TTAGGGCATCAGGAAACAGTTGGTGAAGTAGGAGGATCTTCTCCAGCTATGGAGGAGAGAACAGGGGTTGAAACTGCTGGAAGAACATCTCTATCAGAAAATCCTC : 1060
 L G H Q E T V G E V G G S S P A M E E R T G V E T A G R T S L S E N P

TTGTCAATGAGATAGTTCAGTCTACTGCATCTGCATTTGACGAACAAAGTATCAAGGATTACTCGTCTAAGATTTCTAATGAAGAAAACCAAGGAAATCATGAAAC : 1166
 L V N E I V Q S T A S A F D E Q S I K D Y S S K I S N E E N Q G N H E

CTTGCCTTCTGTTGAAGAATCTAAAAGGATACCAGAGAATAATGCAGAGAACAAGGAAACCAATCAGATTGCTGAAGAACAGAAACGTGAGCCTGTTTCCTCATCT : 1272
 T L P S V E E S K R I P E N N A E N K E T N Q I A E E Q K R E P V S S S

                                                                   <--------------
GTTGCTGCTAGCACCCCTCTTGTTCGTCCCGCTGGCCTTGGATCTGCAGCTCCGTTGTTGGAACCGGCTGCCCGGGTGGTGCAGCAGAGCCTCGGGTGAACTATAC : 1378
V A A S T P L V [R P A G L G S A A P L L E P A A R V] V Q Q P R [V N Y T

TGTATCTGATACACAACCCCGAAAAACAGAAGACTCCTCAATTGGGGAGGCTGATGAGTATGACGAGACTCGAAGAAAAACTTCAAA : 1465
V S D T] Q P R [K T E D S S I G E A D E Y D E T R R] K T S
```

Supplemental Figure 3

SUPPLEMENTAL FIGURE 3 | Sequence of Toc120 A-domain as determined by 5'-RACE PCR. The peptides from the MS results were used to design degenerate oligo nucleotides, which were applied in a 5' RACE-PCR approach (arrow). The obtained cDNA was cloned and sequenced. The arrow head indicates the designated start methionine.
