# Supplementary material for: A new member of the psToc159 family contributes to distinct protein targeting pathways in pea chloroplasts
Source: Front Plant Sci. 2014 May 28;5:239. doi: 10.3389/fpls.2014.00239 (PMC4036074; doi:10.3389/fpls.2014.00239)
Supplement: Supplementary file 6 [file Presentation4.PDF]

AAGCAGTGGTATCAACGCAGAGTACGCGGGAATGAAGAAACCGAGGATGATGATGCCAGATCAAAGTCAGAGCATCTGGAAACAATTGGTGAAGCCGGAGGATCTTCTCTTGCTGTG : 117

K Q W Y Q R R V R G N E E T E D D D A R S K S E H L E T I G E A G G S S L A V

GATGAAAACAAAGTGATTGAAACTGCTGGAAGCTCATCCCTTTCAGAAATTTCTTTTGCTAATCAGATTCCGGCTGTTCAGGATACTGCAGCTGATTCAGAAGAAGGGAGTGCAAAG : 234

D E N K V I E T A G S S S L S E I S F A N Q I P A V Q D T A A D S E E G S A K

CTTTACCAGTCTCAGATTTCTAAGGCAGAAAATCAAGGAAATTATGAAAACTTATCTGTTGTTGAAAGAAGAAAAGTGATTGAAACAGGAGGATCTTCTCCGGCTTTGGATGAAAGA: 351

L Y Q S Q I S K A E N Q G N Y E N L S V V E R R K V I E T G G S S P A L D E R

ACAGTGACTGAAACTATTGGAAGCTCATCTCCACCAGAAGACTCTTTTGCTAATGAGACTCCGACTGTTCAGGCTACTGCAGCTGAAACAGGAGGGTCTTCTCTGGCCTTGGACGAA : 468

T V T E T I G S S S P P E D S F A N E T P T V Q A T A A E T G G S S L A L D E

AGAGCAGTGACTGAAACTGTTGGAAGCTCATCTCCATCAGAAAAATCTTTTGCTAATGAGACGCTGATTATTCAGGCTACTGCAGCTGAAACAGGAGGATCTTCTCCGGCCTTGGAC : 585

R A V T E T V G S S S P S E K S F A N E T L I I Q A T A A E T G G S S P A L D

GAAAGAGCAGTGACTGAAACTGTTGGAAGCCCATCTCCATCAGAAAAATCTTTTGCTAATGAGATGCCAACTGTTCAGGCTGCTGCAGCTGATCCAGAAGAGGGGAGTACAAAAGTT : 702

E R A V T E T V G S P S P S E K S F A N E M P T V Q A A A A D P E E G S T K V

TACTTGTCTAAGATTTCGAATGAAGAAAAACAAGGAAATTATGAAAAGTCATTTTTCGTTCAGGAACCTGAAAAGATATCAGAGAATAATGCGAAAGAGAAGCAAACTACTCAGATC : 819

Y L S K I S N E E K Q G N Y E K S F F V Q E P E K I S E N N A K E K Q T T Q I

ACTAAAGAACATGAGCTTGATTCTTTATCTGGAAAACCTGTTGCTACTAGCACCCCTCTTGACCATCCTGTTGGCCTTGGATCTGCAGCTCCATTGTTGAAACCTGCTCCTAGGGTA : 936

T K E H E L D S L S G K P V A T S T P L D H P V G L G S A A P L L K P A P R V

GTGCAGCAGCCAGGGGTGAATAGTATCAAAAAAAAGCAATCCAATCAGATCATTAATAAACATAATAGTGAGTTTGATTCCTCATCTGGAAAATCTGTTGCTGCTAGCACCCCTCTT : 1053

V Q Q P G V N S I K K K Q S N Q I I N K H N S E F D ***S S S G K S V A A S T P L***

GATCGTCCCGTTGGCCTTGAATCTGCAGCTCCATTATTGAGACCTGCTCCTAGGGCTGTGCAGCAACCACGGATGAATAATACTAAAGAAAAGCAAACCAATCAGATCACTAAAGAA : 1170

D R P V G L E S A A P L L R P A P R A V Q Q P R M N N T K E K Q T N Q I T K E

CAGAATAGAGAGCTTGATTCCTCATCTGGACATTCTGTTGCTACTAGCACTCCTCATGTTCGTCCTGTTGACCTTGGACCTGCAACTTCTTTGTTGGAACCTGCTCCCAGGGTAGTG : 1287

Q N R E L D S S S G H S V A T S T P H V R P V D L G P A T S L L E P A P R V V

CAGCAGCCACGGGTGAATAATACTGTTTCTAATACACAGTCCCAAAAAATAGAAGACTCGTCAACTGTGGAGGCTGAGGAGTATGATGAGACTCGAGAGAAACTTCAAATGATTAGG : 1404

Q Q P R V ← PsToc132A (RACE) **T Q S Q K I E D S S T V E A E E Y D E T R E K L Q M I R**

GTGAAGTTTTTGCGGCTAGCTAATAGGCTTGGG : 1437

V K F L R L A N R L G

Supplemental Figure 4

SUPPLEMENTAL FIGURE 4 | Sequence of Toc132 A-domain as determined by 5'-RACE PCR. The putative amino acid sequence of the pea A–domain of Toc132 (PsToc132A) is shown in italics blue. Amino acid sequence of the G-domain is shown in black and the putative sequence amplified via 5'-RACE is shown in italics black. Position of the gene specific primer used in the 5' – RACE id indicated with arrow. The peptides identified by mass spectrometry sequencing are framed.
