# Supplementary material for: A new member of the psToc159 family contributes to distinct protein targeting pathways in pea chloroplasts
Source: Front Plant Sci. 2014 May 28;5:239. doi: 10.3389/fpls.2014.00239 (PMC4036074; doi:10.3389/fpls.2014.00239)
Supplement: Supplementary file 7 [file Presentation5.PDF]

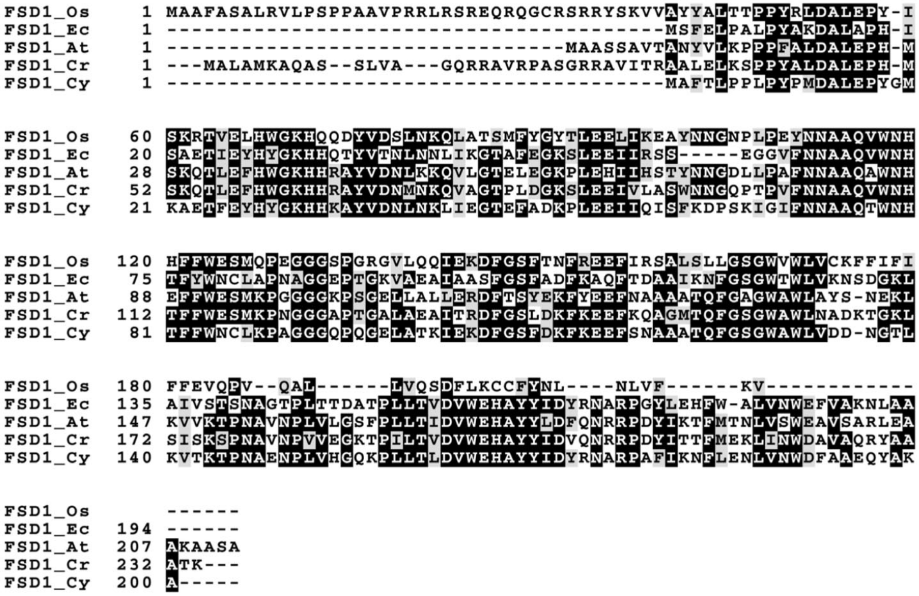

Supplemental Figure 5

SUPPLEMENTAL FIGURE 5 | Alignment of FSD1 proteins from different species. FSD1 protein sequences from At (Arabidopsis thaliana), Os (Oryza sativa), Cr (Chlamydomonas reinhardii), Cy (Cyanobacterium PCC 7702) and Ec (Escherichia coli DH1) were aligned using the Clustal omega program. Amino acids identical in all proteins are shaded in black, amino acids identical in four or less proteins are shaded in grey (BoxShade).
